# Stronger functional connectivity during reading contextually predictable words in slow readers

**DOI:** 10.1038/s41598-023-33231-x

**Published:** 2023-04-12

**Authors:** Kim-Lara Weiss, Stefan Hawelka, Florian Hutzler, Sarah Schuster

**Affiliations:** 1 Department of Psychology, Centre for Cognitive Neuroscience, Paris-Lodron-University of Salzburg, Hellbrunnerstr. 34, 5020 Salzburg, Austria; 2 Uniklinik Köln, Cologne, Germany

**Keywords:** Psychology, Human behaviour

## Abstract

The effect of word predictability is well-documented in terms of local brain activation, but less is known about the functional connectivity among those regions associated with processing predictable words. Evidence from eye movement studies showed that the effect is much more pronounced in slow than in fast readers, suggesting that speed-impaired readers rely more on sentence context to compensate for their difficulties with visual word recognition. The present study aimed to investigate differences in functional connectivity of fast and slow readers within core regions associated with processing predictable words. We hypothesize a stronger synchronization between higher-order language areas, such as the left middle temporal (MTG) and inferior frontal gyrus (IFG), and the left occipito-temporal cortex (OTC) in slow readers. Our results show that slow readers exhibit more functional correlations among these connections; especially between the left IFG and OTC. We interpret our results in terms of the lexical quality hypothesis which postulates a stronger involvement of semantics on orthographic processing in (speed-)impaired readers.

## Introduction

Word predictability is supposed to index top-down processes during reading which facilitates the integration of a words’ meaning and fosters experience-based expectations about upcoming words^1^. The beneficial effect of being able to predict upcoming words has been well-documented in both eye movement studies^2–6^ and neuroimaging studies^7–11^. Top-down processes during reading, however, may not only affect semantic access by activating a words’ meaning^12,13^, but may also impact early visuo-orthographic processing^14–19^. The present study aimed at investigating the functional connectivity which is supposed to be informative of potential top-down guidance to aid visual word recognition.

Evidence supporting the notion that word predictability acts upon visuo-orthographic processing primarily stems from neuroimaging studies reporting early effects of word predictability in occipital areas. To illustrate, Dikker and colleagues^15,16^ investigated the impact of violating syntactic word category predictions using magnetoencephalography (MEG). The authors reported higher activation of the visual cortex within an early time window (i.e., around 120 ms) for syntactically unpredicted word categories (e.g., “The recently princess …”) compared to word categories which did match word category expectations (see also^20^). These results further support the idea that prior context affects the generation of expectations about the visual word form of upcoming words via top-down modulation from higher order language areas to lower-level sensory areas^21^. Furthermore, the left occipito-temporal cortex (OTC)—entailing the supposed “*Visual Word Form Area*”^22–24^—showed a reduced response to predictable compared to unpredictable words in previous studies which, supposedly, emerged from top-down (pre-)activation^18,19,25^.

The beneficial effect of word predictability has been shown to vary between various levels of reading performance, indicating a more pronounced facilitation for slow than for fast readers^26,27^. The study by Ashby et al.^26^ found that proficient readers do not exhibit differences when reading semantically constrained sentences compared to unconstrained sentences. Less proficient readers, on the contrary, relied on context to support processing predictable (low frequency) words when the sentence context allowed them to do so (i.e. in case of semantically constrained sentences). The study by Hawelka et al.^27^ reported an early (but small) effect of word predictability in fast readers and a later but substantially stronger effect in speed-impaired readers. A possible explanation for this finding may be linked to impoverished lexical representations as postulated by the lexical quality hypothesis^28^. This hypothesis suggests a different capacity of lexical representations for fast and slow readers, causing greater reliance on context (i.e., high predictability) to support word processing for the latter group^29^. More precisely, it assumes that efficient skilled reading processes, such as comprehension, are dependent on high-quality word representations “*that include well specified orthographic, phonological and semantic-syntactic information*”^28^, p. 211.

The underspecified lexical representations—in particular the visual-orthographic representations—could be responsible for the poor reading rate in slow readers, as the impaired orthographic representations are preventing fast and efficient bottom-up processing. Note that impaired reading speed in a transparent orthography, such as German, is the cardinal symptom of developmental dyslexia, whereas accuracy and comprehension are usually spared^30,31^. In fast readers, word recognition is considered to proceed from the activation of the orthographic representation towards the activation of phonology and semantics (as a result of their high quality lexical representations), supposedly without any substantial top-down contributions from semantics on orthographic processing (but see^32^). In slow readers this “context-free” bottom-up decoding might be bottle-necked by less specified (“lower quality”) visual-orthographic representations. As a consequence, the beneficial effect of word predictability caused by top-down, context-based processing has the potential to exert its influence^27^. Thus, for slow readers one would expect a stronger involvement of higher-order language processing, such as semantic processing, on visuo-orthographic processing.

In the present study, we aim to bring together the assumptions of the lexical quality hypothesis with insights gained from neuroimaging. In a sample of fast and slow readers we investigate the functional connectivity between brain regions exhibiting a sensitivity to word predictability^18,19,25,33^. Thus, functional connectivity was assessed by means of correlating the neuronal activation between regions of interest (ROI-to-ROI correlations) in a supposedly domain-specific network^34^ implicated in higher-order language (e.g., semantic), visuo-orthographic and visual processing^35–39^. Specifically, for the higher-order language nodes we were interested in the correlated response of the left middle temporal gyrus (MTG) and inferior frontal gyrus (IFG). The left OTC served as our ROI indexing visuo-orthographic processing^23,24^ along with a region in the early visual cortex. Critically, not only are these target regions well documented in terms of regional activity, but various findings demonstrated crucial structural connections enabling between areal communication^40,41^. Following the lexical quality hypothesis, we hypothesize that the functional connectivity between the OTC and MTG and/or IFG is stronger in slow than in fast readers. Put differently, slow readers should exhibit a stronger functional connectivity between the orthographic and higher-order language nodes corroborating the notion of stronger contributions from semantics onto (less specified) visuo-orthographic representations.

## Methods

We re-analyzed combined fMRI and eye-tracking data from a previous study of our lab^18^. In the following we will briefly describe the used material, procedure, data acquisition and statistical analysis. For an in-depth description see^18^.

### Participants

A total of 56 (31 male) undergraduate students participated in the study (mean age = 25 years; *SD* = 5 years). All participants reported that they had no history of neurological or psychiatric disorders and that they have normal or corrected-to-normal vision. Their reading speed was assessed by means of two measures: (i) the words per minute read during the course of the experiment and (ii) a standardized reading speed test which is currently being developed in our lab (for a computerized version see^42^). The reading speed test required judging the meaningfulness of sentences within a time limit of 3 min (i.e., judging the semantic correctness of sentences). Since judging the meaningfulness of the sentences is very easy (less than 1% erroneous responses), the number of correctly marked sentences can be considered as a measure of reading speed. The correlation between the outcome of this paper–pencil test and the words per minute read during the experiment was *r* = 0.68. A subset of the speed-impaired participants self-reported a history of reading difficulties (*n* = 12; ~ 21%) and some had a diagnosis of dyslexia at the time of their formal education (*n* = 8; ~ 14%). Functional connectivity analyses focusing on this “clinical” subsample are presented in the Supplementary Material (Fig. S1).

To differentiate between fast and slow readers, we used the above-mentioned measure of words per minute (for a similar approach see^27^). Since the study was conducted in German (i.e., a transparent orthography), we considered low reading speed as an indication of deficient lexical representations. Specifically, in a language with a reliable grapheme-phoneme correspondence (such as German) reading speed impairments can be considered the core deficit of dyslexia^43,44^, as opposed to opaque orthographies (such as English) in which reading accuracy is also affected.

For our functional connectivity analysis in slow and fast readers, we split our sample on the basis of the median of our words per minute measure. This procedure resulted in our final groups of slow and fast readers (both *n* = 28), exhibiting a mean wpm of 166 (*SD* = 30) and 263 (*SD* = 36), respectively. Before scanning, participants gave their written informed consent. The experiment was conducted in accordance with the Declaration of Helsinki and was approved by the local ethics committee of the University of Salzburg.

### Material

Participants silently read 117 sentences from the Potsdam Sentence Corpus^3^ for comprehension. The sentences were all grammatically and semantically legal and cover a variety of typical German sentence structures. For the analysis, we excluded the first word of each sentence which left us with a total of 771 words for the analyses. The Potsdam Sentence Corpus provides predictability estimates for each and every word of the sentences based on an independent norming sample (*n* = 82 complete protocols^3^). These norms range between 0 and 1 denoting completely unpredictable and (completely) predictable words, respectively (*M* = 0.21; *SD* = 0.28). For all analyses (behavioral and neuroimaging), we *logit*-transformed the word predictability norms as in the original reports^3,4^. In addition to word predictability, we further considered word frequency which we derived from the CELEX database^45^ (i.e., *log*-transformed occurrences per million; range: 0.0–4.4; *M* = 2.1; *SD* = 1.3) and word length (*M* = 5.5; *SD* = 2.4) in the analyses (see below).

### Procedure

Sentence presentation was preceded by the appearance of two fixation-bars at the vertical center near the left border of the screen. The bars remained on screen for a variable time ranging from 1000 to 3000 ms (with increments of 500 ms). Thereafter, a sentence appeared in the horizontal center of the screen. Fixating a cross at the bottom of the right corner of the screen terminated the sentence presentation. After, on average, every 10% of the sentences, participants had to answer (via button press) a simple two-alternative forced-choice question with regard to the content of the preceding sentence (12 questions in total). During 24 null-events the fixation-bars remained on the screen for additional 2 s.

### Data acquisition and analysis

#### Eye-tracking

Eye movements were recorded monocular with an EyeLink CL system in the long-range setup (SR-Research, Ontario, Canada) with a sampling rate of 1 kHz. The camera was placed at the rear end of the scanner bore at a distance of approximately 90 cm behind the participant and approximately 120 cm in front of the screen. A horizontal three-point calibration routine preceded each of the three scanning sessions. Each trial was preceded by a drift correction/fixation control procedure in which a fixation had to be detected by the eye-tracking system between the fixation bars*.* In case the control procedure failed, the system was re-calibrated.

We analyzed our eye movement data by means of linear mixed models (LMM) with the *lme4*-package (version 1.1-12^46^) running in the R environment for statistical computing (version 3.6.0^47^). As the dependent variable for this analysis, we used participants’ gaze duration which is the sum of all fixation durations on a word during first-pass reading. Participants (*sbj*), items (*wrd*) and sentences (*sen*) were treated as random effects, whereas *group* (fast versus slow readers; *grp*), word predictability (*prd*), word frequency (*frq*), (reciprocal) word length (*len*) and relative word position (*pos*) as fixed effects. Furthermore, we included the interaction between frequency and predictability, group and frequency, and group and predictability into the model in accordance with previous reports^27^. The formula of the model was as follows: *log(gaze)* ~ *grp* + *prd* + *frq* + *len* + *pos* + *grp:prd* + *grp:frq* + *frq:prd* + (1|*sbj*) + (1|*wrd*) + (1|*sen*). The data as well as the corresponding code for this (and subsequent) analyses are publicly available and can be found under the following link: https://osf.io/cgj79/. Note that fixations shorter than 80 ms were excluded from both the eye-tracking and fMRI analysis.

#### Image acquisition

Functional imaging data were recorded with a Siemens Magnetom Trio 3 Tesla scanner (Siemens AG, Erlangen, Germany) equipped with a 12-channel head-coil. Functional images sensitive to BOLD contrast were acquired with a T2*-weighted gradient echo EPI sequence (TR 2000 ms, TE 30 ms, matrix 64 × 64 mm, FOV 192 mm, flip angle 80°). Thirty-six slices with a slice thickness of 3 mm and a slice gap of 0.3 mm were acquired within the TR. Scanning was divided in 3 sessions with a variable number of scans per session. The exact number of scans depended on the participants’ reading speed and potential re-calibration procedures and ranged from 106 to 437 scans (*M* = 152; *SD* = 39 scans). In addition to the functional images, a gradient echo field map (TR 488 ms, TE 1 = 4.49 ms, TE 2 = 6.95 ms) and a high resolution (1 × 1 × 1.2 mm) structural scan with a T1-weighted MPRAGE sequence were acquired from each participant.

#### fMRI data analysis

For preprocessing and statistical analysis of the fMRI data we used SPM12 software (http://www.fil.ion.ucl.ac.uk/spm/) running in a MATLAB 8.1 environment (Mathworks Inc., Natick MA, USA). Functional images were corrected for geometric distortions with the FieldMap toolbox, realigned and unwarped, and then coregistered to the high resolution structural image. The structural image was normalized to the MNI T1 template image, and the resulting parameters were used for normalization of the functional images, which were resampled to isotropic 3 × 3 × 3 mm voxels and smoothed with a 6 mm FWHM Gaussian kernel. No slice timing correction was applied.

Statistical analysis was performed by computing a fixed effects model on the first level and a random effects model on the second level. The BOLD response was related to the eye-tracking data in the specifications of the subject-specific first level model: each onset of a first fixation on a word was used in an onset vector to model the canonical hemodynamic response function. First fixation onsets on the first word of each sentence as well as the onsets and durations of the comprehension questions were not analyzed further, but coded in separate onset vectors of no interest along with six head movement parameters derived from the preprocessing. The functional data of these first level models were high-pass filtered with a cutoff of 128 s and corrected for autocorrelation by an AR(1) model^48^. Parameter estimates of the first level models were further calculated in the context of a General Linear Model (GLM^49^). Word predictability, word frequency and word length were added as parametric regressors of the reading versus baseline (i.e., including interstimulus intervals, null-events, and drift correction/re-calibration procedures) contrast. Furthermore, orthogonalization was deactivated in the single subject analyses which ensures that the present results capture the unique variance assigned to each of the parametric regressors^50^. The resultant subject-specific contrast images for word predictability were then used for the second level random effects analysis and submitted to one-sample *t*-tests. Statistically significant effects on the whole-brain level were identified using a voxel-level threshold of *p* < 0.005 (uncorrected) and a cluster-level threshold of *p* < 0.05 (FDR-corrected for multiple comparisons).

#### Definition of regions of interest (ROIs)

Based on our group results presented in Fig. 2 and reported in Table 2, we defined five left-lateralized regions of interest, that are (i) the occipital pole (OCC; *x* = − 21, *y* = − 91, *z* = − 11), (ii) the occipital-temporal cortex (OTC *aka* fusiform gyrus; *x* = − 39, *y* = − 67, *z* = − 17), (iii) the posterior middle temporal gyrus (pMTG; *x* = − 48, *y* = − 37, *z* = 1), (iv) the anterior middle temporal gyrus (aMTG; *x* = − 57, *y* = − 7, *z* = − 11) and (v) the triangular part of the inferior frontal gyrus (IFG; *x* = − 54, *y* = 26, *z* = 4). Based on the respective group coordinates, we extracted signal change estimates (using spheres of 3 mm radius).

## Results

### Behavioral results

**Figure 1:**
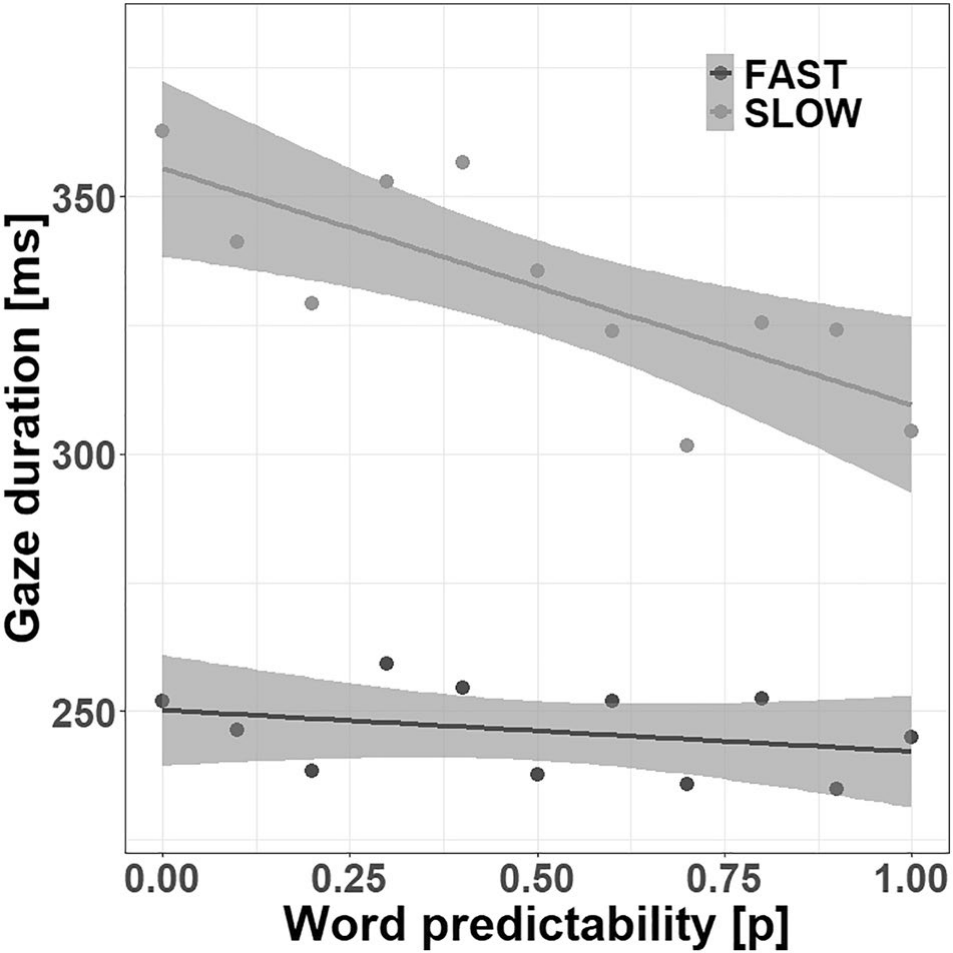
The effect of word predictability for fast and slow readers on their gaze duration. The points represent the average gaze duration over the rounded predictability values from 0 to 1 (with increments of 0.1). The shaded areas denote 95% pointwise confidence intervals.

The analysis of the two-alternative forced-choice questions revealed a minimum of 11 (out of 12) correct answers. The group of the slow and the fast readers did not differ in this regard, *t* < 1. As aforementioned, we opted for participants’ gaze duration (*log*-transformed) as the dependent measure for our linear mixed model (LMM) analysis which is defined as the sum of all fixation durations on a word during first-pass reading and can be seen as a proxy of visual word recognition^51^. The results of the LMM analysis are provided in Table 1. Central for the present study, we observed an interaction between group and word predictability which is illustrated in Fig. 1. As depicted, apart from the large group effect, the slow readers exhibited a much more pronounced effect of word predictability than the fast readers (54 ms versus 10 ms).

**Table 1:** Fixed effects, standard errors (SE) and corresponding test statistics of the LMM on participants’ gaze duration.

	Estimate	SE	*t*-value
(Intercept)	5.481	0.038	144.22
Group	0.328	0.039	8.46
Length	− 0.967	0.080	− 12.12
Frequency	− 0.001	0.008	− 0.07
Predictability	− 0.030	0.008	− 3.59
Relative word position	0.189	0.019	9.90
Group × frequency	− 0.030	0.004	− 7.16
Group × predictability	− 0.015	0.004	− 3.51
Length × frequency	0.011	0.003	3.70

### fMRI results

#### Effects of word predictability

**Figure 2:**
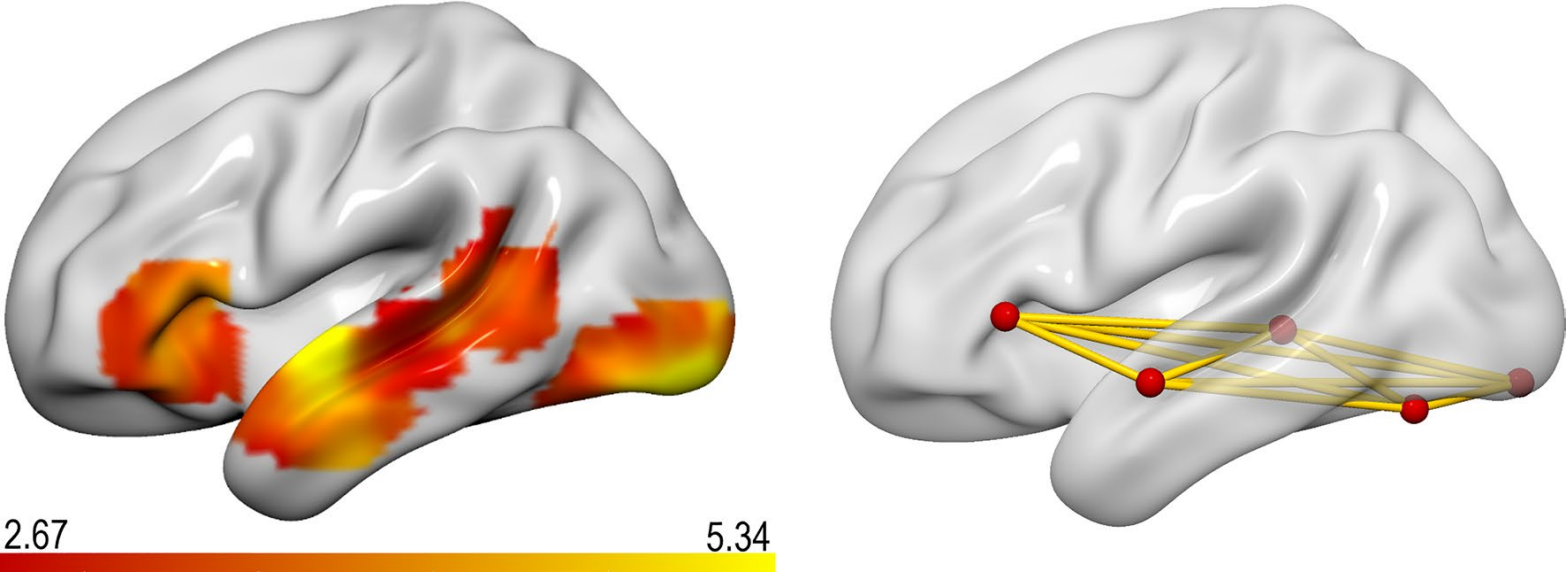
The left panel illustrates those brain regions, which showed a decrease in activation for words with increasingly higher predictability. The right panel illustrates the location of the selected ROIs for the functional connectivity analysis. Images were generated using the *Surf Ice* tool (version v2.1.51–0) for visualizing connectome networks, tractography and statistical maps (https://www.nitrc.org/projects/surfice/).

As can be seen in Table 2 and Fig. 2, word predictability was associated with a decrease in activity in bilateral occipital regions, extending in the left hemisphere into more anterior proportions of the left occipito-temporal cortex. Furthermore, we observed this negative linear association within the left anterior-to-posterior middle temporal gyrus and surrounding regions, as well as the triangular part of the left inferior frontal gyrus. The reverse contrast, that is, increasing activity with increasing word predictability did not reveal any significant clusters. These observations replicate previous findings from our lab on the word predictability effect^18,25,52^.

**Table 2:** Regions exhibiting an effect of word predictability.

Region	Voxel extent	MNI coordinates			*t*
		x	y	z	
L occipital pole	151	− 21	− 91	− 11	5.34
L occipital fusiform gyrus		− 39	− 67	− 17	2.89
R inferior occipital gyrus	92	33	− 88	− 8	4.60
R occipital fusiform		39	− 73	− 11	3.89
R occipital pole		24	− 94	− 8	3.59
L anterior middle temporal gyrus	67	− 57	− 7	− 11	4.31
L inferior frontal gyrus triangular part	65	− 54	26	4	4.05
L posterior middle temporal gyrus	63	− 48	− 37	1	3.69
L occipital pole	151	− 21	− 91	− 11	5.34
L occipital fusiform gyrus		− 39	− 67	− 17	2.89
R inferior occipital gyrus	92	33	− 88	− 8	4.60
R occipital fusiform		39	− 73	− 11	3.89
R occipital pole		24	− 94	− 8	3.59

*L* left hemisphere, *R* right hemisphere.

#### Group differences

Comparing the effect of word predictability between fast and slow readers revealed no statistically significant differences in either direction at the level of regional activity (i.e., fast > slow; slow > fast readers). This observation stands in marked contrast to the results of our behavioral analysis. In the following we report results from a functional connectivity analysis to further investigate the neuronal correlates of reading speed related word predictability effects.

#### Functional connectivity

**Figure 3:**
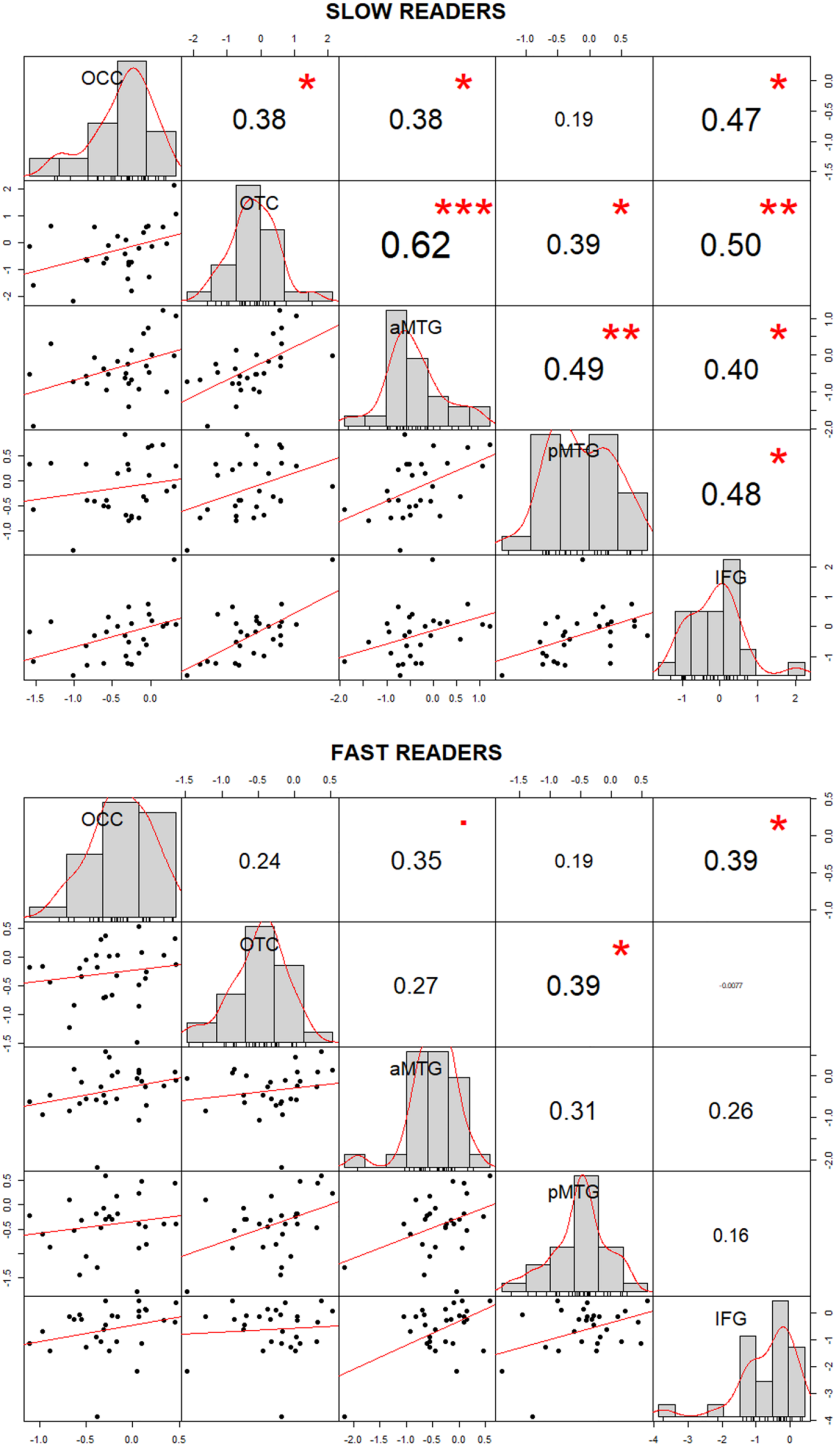
Scatter plots, histograms and correlation coefficients of our ROI-to-ROI analysis in slow (upper panel) and fast readers (lower panel). Significant spearman rank correlations are marked by asterisks: *p < 0.05, **p < 0.01, ***p < 0.001.

**Figure 4:**
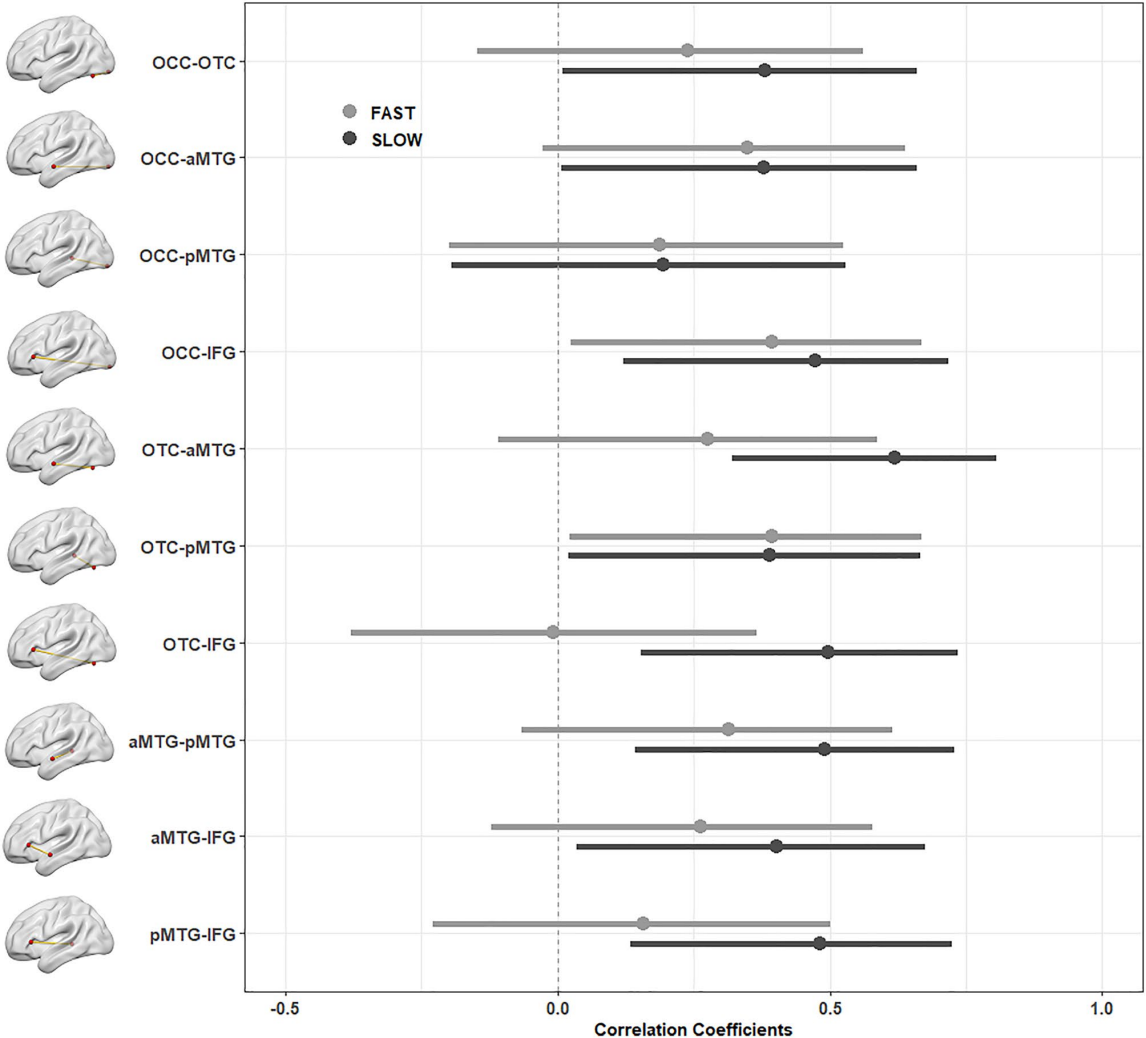
Correlation coefficients (with 95% confidence intervals) for all connections among our ROI-to-ROI analysis for fast (light grey) and slow (dark grey) readers.

The results of our ROI-to-ROI correlational analysis are presented in Fig. 3. Within our network of selected ROIs (illustrated in Fig. 2), we observed moderate correlations in our sample of fast readers between the IFG ↔ pMTG/aMTG, the pMTG ↔ aMTG/OTC connections. In our sample of slow readers, by contrast, we observed moderate to high correlations between almost all defined connections (apart from the pMTG ↔ OCC connection). Comparing these Spearman rank correlations among fast and slow readers revealed a significant difference in the IFG ↔ OTC connection as assessed by Fisher Z-transforming the correlation coefficients, *Z* = 1.96, *p* < 0.05 (Bonferroni-corrected for multiple comparisons). Figure 4 illustrates group-specific coefficients with their respective confidence intervals.

## Discussion

The present study aimed to connect core assumptions of the lexical quality hypothesis, which is formulated purely cognitive in nature, with findings from neuroimaging. In short, the lexical quality hypothesis assumes that fast and efficient skilled reading is dependent on high quality word representations^28^. As a consequence, the poor reading rate in slow readers could be explained by underspecified (“low quality”) lexical representations. On the contrary, in fast readers word recognition is considered to proceed from the activation of the orthographic representation towards the activation of phonology and semantics supposedly without any substantial top-down contribution. If this “context-free” decoding is prevented in slow readers, we would expect a stronger involvement of semantic processing on orthographic processing as the beneficial effect of word predictability caused by top-down, context-based processing has the potential to exert its influence (see^27^).

To address this assumption, we studied the effect of word predictability by investigating the correlated neural response among previously identified brain regions associated with processing predictable words, that is, the left occipito-temporal cortex (OTC), middle temporal (MTG) and inferior frontal gyrus (IFG) in fast and slow readers. As target regions for our functional connectivity analysis, we identified the left MTG and IFG (as potential sources of top-down activation), the left OTC and occipital cortex (OCC) as visual and visuo-orthographic bottom-up processing nodes. We hypothesized that slow readers would exhibit a stronger functional connectivity between the orthographic and higher-order language (e.g., semantic) nodes corroborating the notion of stronger contributions onto (less specified) visuo-orthographic representations.

In line with previous studies from eye movement research, we observed an effect of word predictability with shorter fixation durations for predictable compared to unpredictable words^2–6^. More crucially, our findings revealed an interaction between word predictability and reading speed. Fast readers showed a slight effect of word predictability on their fixation durations, whereas slow readers massively benefited from word predictability^26,27^. However, it has to be noted that fast readers tend to skip predictable words, while slow readers, who generally show an extremely low skipping rate, seem to process those words with a single fixation and, thus, contribute more observations to the effect of word predictability on fixation durations^27,53^.

With respect to our general linear model (GLM) analysis investigating regional activation related to word predictability, we observed a decrease in activation with increasing predictability in the bilateral occipital regions extending anteriorly to the left fusiform gyrus, in the left anterior-to-posterior MTG and the triangular part of the left IFG which is in line with previous research^18,19,25,33,52^. This orchestration of regional activity has been associated with facilitated retrieval of lexico-semantic information as well as residual prediction error processing^18,19,25^. Surprisingly and at odds with the behavioral findings, we found no group differences related to the effect of word predictability in our GLM analysis.

Our ROI-to-ROI correlation analysis, however, revealed that slow readers exhibit a more pronounced functional connectivity among core regions of the brains’ reading network (as identified in our GLM analysis) than the fast readers. That is, our fast readers show correlated responses within higher-order language regions, while slow readers additionally show synchronized activity within regions associated with visual and visuo-orthographic processing. Especially, the connection between the OTC and IFG was markedly distinct among our samples of fast and slow readers. The left OTC is assumed to host the so-called “Visual Word Form Area” (VWFA;^22^) which has been shown to play an essential part in orthographic decoding^23,24^. Crucially, previous fiber-tracking studies demonstrated that the left OTC (including the VWFA) shows strong anatomical connections with the perisylvian language areas via several fasciculi such as the inferior longitudinal fasciculus (ILF), the inferior fronto-occipital fasciculus (IFOF), the vertical occipital fasciculus (VOF) and the arcuate fasciculus (AF) targeting superior temporal regions^40,54–58^. Probing the directed connectivity by means of dynamic causal modeling (DCM) between the left OTC and IFG, Woodhead and colleagues^59^ demonstrated that the left IFG modulates the activation in the left OTC within early stages of word processing (i.e., around 200 ms) supporting the assumption of top-down processing from higher-order language areas on orthographic processing. Our findings show that this connection is functionally stronger synchronized in slow readers. This may indicate that they harness top-down processes to compensate for poor bottom-up decoding.

Interestingly, three out of four connections with the early visual processing node (i.e., the OCC) showed a synchronized response in slow readers. We speculate that this finding may indicate a stronger involvement of top-down processes upon early visual processing as has been proposed in the context of the predictive coding framework^60–62^. In short, it has been argued that our brain—as a proactive organ—continuously forms top-down predictions about upcoming sensory events. In our case, such sensory events represent visual features of expected word forms which are being top-down activated. In fast readers this may occur before the word is actually fixated^4,27^. Such a “pre-sensitizing” of lower cortical regions would explain why less neural energy is needed to represent those visual features at the time of the actual encounter^14,17^. In slow readers, this mechanism seems to be more pronounced (but also belated compared to fast readers^27^). A putative explanation could be as follows. Slow readers rely on a smaller “grain size” (sublexical units) for visual word recognition^63^. It may be that they use a word’s initial sublexical unit (e.g. the first syllable^64^) as an access unit to the phonological (and subsequently the semantic) lexicon. That means that a (contextually fitting) lexical representation is activated prior to the instantiation of the visuo-orthographic whole-word template (in the orthographic lexicon). This instantiation of the whole-word orthographic unit may be accomplished with top-down aid.

## Limitations

Our study has some limitations. First, we used functional connectivity to assess synchronized responses for which we can only speculate about directionality. Future studies may investigate the cortical coupling between hubs of the reading network with effective connectivity analyses to ascertain their directed influence in fast and slow readers^65^. Second, the present study applied a quasi experimental approach, that is, we used sentences from a corpus which aimed at capturing the whole spectrum of word predictability^3,4^. Thus, we could not employ a classical factorial design contrasting (completely) unpredictable with highly predictable words. Third, we treated reading speed as a dichotomous trait and in our group of slow readers we mixed garden-path slow readers with diagnosed dyslexics. Future studies may treat reading speed as continuous or make a clear distinction between normal and dyslexic readers. Note, however, that a separate analysis with only those readers who had a formal diagnosis of developmental dyslexia (see Supplementary Material) revealed similar findings as for the whole group of slow readers.

## Supplementary Information

Supplementary Figure S1.

## Data Availability

Code and data are available under the following link: https://osf.io/cgj79/.
