# Supplementary material for: Stronger functional connectivity during reading contextually predictable words in slow readers
Source: Sci Rep. 2023 Apr 12;13:5989. doi: 10.1038/s41598-023-33231-x (PMC10097649; doi:10.1038/s41598-023-33231-x)
Supplement: Supplementary file 1 — Supplementary Figure S1. [file 41598_2023_33231_MOESM1_ESM.docx]

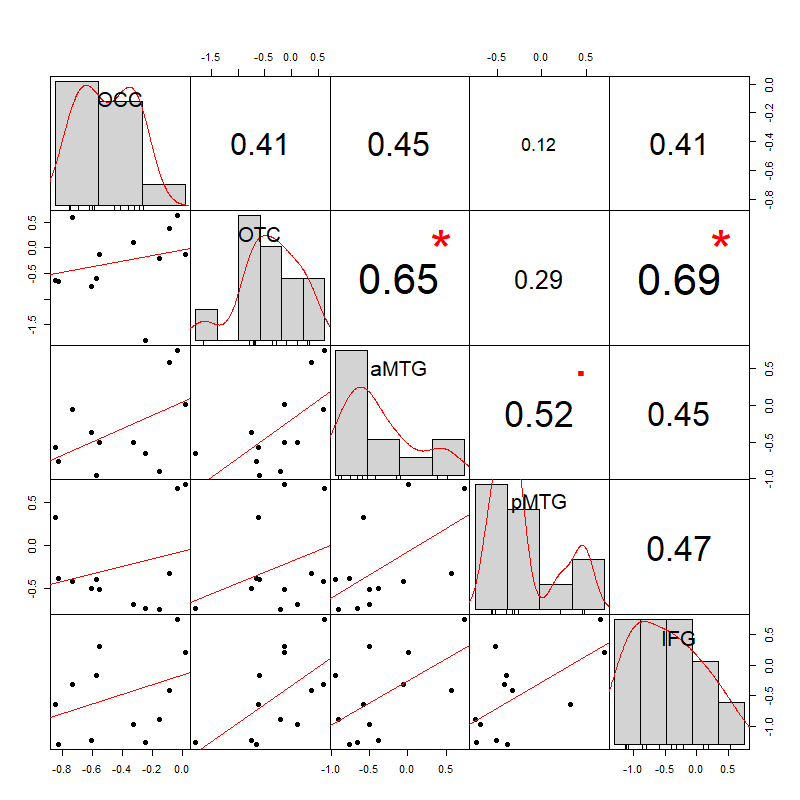


*Figure S1*. ROI-to-ROI correlation (i.e. Spearman's rank correlation) of the subsample of participants reporting a persistent history of reading difficulties (*n* = 12), with some of them even being diagnosed with dyslexia during their formal education (*n* = 8).
